# Predicting breast cancer drug response using a multiple-layer cell line drug response network model

**DOI:** 10.1186/s12885-021-08359-6

**Published:** 2021-05-31

**Authors:** Shujun Huang, Pingzhao Hu, Ted M. Lakowski

**Affiliations:** 1 College of Pharmacy, University of Manitoba, Apotex Centre, 750 McDermot Avenue, Winnipeg, Manitoba R3E 0T5 Canada; 2 Department of Biochemistry and Medical Genetics, University of Manitoba, Room 308 - Basic Medical Sciences Building, 745 Bannatyne Avenue, Winnipeg, Manitoba R3E 0J9 Canada; 3 Cancer Care Manitoba Research Institute, 675 McDermot Avenue, Winnipeg, Manitoba R3E 0V9 Canada

**Keywords:** Breast cancer, Drug response, Network model, Data integration

## Abstract

**Background:**

Predicting patient drug response based on a patient’s molecular profile is one of the key goals of precision medicine in breast cancer (BC). Multiple drug response prediction models have been developed to address this problem. However, most of them were developed to make sensitivity predictions for multiple single drugs within cell lines from various cancer types instead of a single cancer type, do not take into account drug properties, and have not been validated in cancer patient-derived data. Among the multi-omics data, gene expression profiles have been shown to be the most informative data for drug response prediction. However, these models were often developed with individual genes. Therefore, this study aimed to develop a drug response prediction model for BC using multiple data types from both cell lines and drugs.

**Methods:**

We first collected the baseline gene expression profiles of 49 BC cell lines along with IC_50_ values for 220 drugs tested in these cell lines from Genomics of Drug Sensitivity in Cancer (GDSC). Using these data, we developed a multiple-layer cell line-drug response network (ML-CDN2) by integrating a one-layer cell line similarity network based on the pathway activity profiles and a three-layer drug similarity network based on the drug structures, targets, and pan-cancer IC_50_ profiles. We further used ML-CDN2 to predict the drug response for new BC cell lines or patient-derived samples.

**Results:**

ML-CDN2 demonstrated a good predictive performance, with the Pearson correlation coefficient between the observed and predicted IC_50_ values for all GDSC cell line-drug pairs of 0.873. Also, ML-CDN2 showed a good performance when used to predict drug response in new BC cell lines from the Cancer Cell Line Encyclopedia (CCLE), with a Pearson correlation coefficient of 0.718. Moreover, we found that the cell line-derived ML-CDN2 model could be applied to predict drug response in the BC patient-derived samples from The Cancer Genome Atlas (TCGA).

**Conclusions:**

The ML-CDN2 model was built to predict BC drug response using comprehensive information from both cell lines and drugs. Compared with existing methods, it has the potential to predict the drug response for BC patient-derived samples.

**Supplementary Information:**

The online version contains supplementary material available at 10.1186/s12885-021-08359-6.

## Background

One of the key goals of precision medicine in breast cancer (BC) is to predict how a cancer patient will respond to a particular chemotherapy or targeted therapy, which could help clinicians prescribe the most effective and least toxic therapeutic strategy. To this end, researchers have been developing computational models to predict the anti-cancer drug response of cancer cells based on their molecular profiles (especially gene expression profiles) using cell line-derived (in vitro) datasets. Ideally, a drug response prediction model should be first trained using existing patient-derived (in vivo) data and then used to predict the response of new patients to a particular drug. However, currently available in vivo datasets, such as The Cancer Genome Atlas (TCGA) [1], do not have enough drug response data to train the drug response prediction models whereas in vitro datasets such as the Genomics of Drug Sensitivity in Cancer (GDSC) [2, 3] and the Cancer Cell Line Encyclopedia (CCLE) [4, 5], provide the response data of hundreds of cell lines to many drugs. Therefore, given the available datasets, cancer cell line-derived datasets provide an alternative method of training in silico drug response prediction models. However, such models should be augmented with drug property data and must eventually be validated with BC patient drug response data.

Many computational models have been developed to predict anti-cancer drug response using cell lines from various cancer types and these models are known as pan-cancer prediction models [6]. Most of these models have been built using the gene expression profiles of cancer cells as input, which have been shown to be the most informative data for drug response prediction in cancer research [3, 7–9]. For instance, Dong et al. [10] developed a support vector machine model to predict the response of various cancer cell lines to particular drugs. The model was trained on the CCLE dataset and used gene expression data as input features. Other types of data, such as gene mutation, copy number variation (CNV), have also been incorporated into prediction models to improve predictive power [3]. For example, ﻿Sharifi-Noghab et al. [11] proposed a deep learning-based model to take gene somatic mutation, CNV, and gene expression data of a particular cell line as input, and predict the response of the cell line to a given drug as the output. In addition, the physical, chemical, and pharmacological properties of drugs such as chemical structure, aqueous solubility, and potency (IC_50_) also play important roles in drug response prediction. Thus, computational models combining genomic profiles with information about the drug’s chemical structure would improve drug response prediction in vitro and in vivo [12, 13]. Menden et al. [12] developed a neural network model which took mutation, CNV and microsatellite instability data of cell lines together with chemical properties of drugs as inputs to predict drug response in the GDSC dataset. Zhang et al. [14] proposed a ﻿dual-layer network, which integrated a cell line similarity network based on gene expression profiles and drug similarity network based on drug chemical structures. Very recently, Wei et al. [15] proposed a new ﻿dual-layer network model, which captures different contributions of all available cell line-drug responses through cell line similarities and drug similarities. The model was applied to CCLE and GDSC independently and demonstrated better performance than some existing studies including the Zhang et al. study [14]. We note that in Wei et al.’s study [15]: 1) the similarity between cell lines was only based on their gene expression profiles while the other omics data types were ignored; 2) the relationships among genes were also overlooked; 3) the similarity between drugs was based on their chemical structures while the other data types, such as drug targets, were not taken into consideration.

More importantly, the models mentioned above were trained only on a per-drug and pan-cancer basis, but they do not take the heterogeneity of cancer types into consideration. Thus, new efforts have been focused on making predictions for drugs for a specific cancer type and are referred to as cancer-specific response prediction models [6]. Some BC-specific models have been developed. The most well-known work is the NCI-DREAM Drug Sensitivity Prediction Challenge [16], which provided drug sensitivity data screened on BC cell lines and along with molecular profiles of BC cell lines to the participants. It aimed to predict drug sensitivity in BC cell lines by integrating multiple-omics data. Forty four drug response prediction models were submitted to the NCI-DREAM Drug Sensitivity Prediction Challenge and the Bayesian multitask multiple kernel learning method demonstrated the best performance [16]. We note that these approaches overlooked the multivariate relationships among genomic features and ignored the fact that functionally similar cell lines may have similar therapeutic response to drugs. In addition, these BC-specific models did not take the information from drugs into consideration and ignored the fact that functionally similar drugs may have similar drug responses on the tested cell lines [16, 17].

In the present study, inspired by Wei et al.’s dual-layer network model [15] and the NCI-DREAM Drug Sensitivity Prediction Challenge [16], we constructed several multiple-layer cell line-drug response network (ML-CDN) models focusing on BC. In the ML-CDN modeling method, cell line similarity networks (CSNs) were first constructed using either one or all of three types (i.e., layers) of molecular profiles: pathway activity profiles, CNV, and mutation profiles. In parallel, drug similarity networks (DSNs) were constructed using either one or all of three drug data types: structures, targets, and pan-cancer sensitivity profiles. Next, each of the CSNs and each of the DSNs were connected by linking the cell lines in the first network to their corresponding (previously tested) drugs in the second network. In the end, a final ML-CDN was selected to predict anti-cancer drug response of TCGA BC patients by estimating the similarities between these patients with BC cell lines.

## Methods

### Data resources and preprocessing

We used the GDSC release 7.0 dataset (ftp://ftp.sanger.ac.uk/pub4/cancerrxgene/releases/release-7.0) as a benchmark dataset in this study, which consists of 1065 cancer cell lines and 266 tested drugs. We downloaded the gene expression, CNV and mutation profiles for 49 BC cell lines. For gene expression data, we used the Robust Multichip Average (RMA) [18] to normalize baseline expression profiles (i.e. coming from untreated samples) for all the BC cell lines. For gene mutation data, the list of genomic somatic variants found in BC cell lines by whole exome sequencing were downloaded and further described as binary features (1 = mutation and 0 = wild type). A gene mutation is annotated if a sequence variation (changes in the protein sequence, e.g. non-synonymous single nucleotide polymorphism) is detected while a gene for which a mutation is not detected in a given cell line is annotated as wild type. CNV data for all genes across all BC cell line samples derived from the Predict Integral Copy Numbers In Cancer analysis [19] of Affymetrix SNP6.0 segmentation data were downloaded and further treated as binary features (1 = amplification or deletion and 0 = wild type). For a gene to be classified as amplified, the entire coding sequence must be contained in one contiguous segment defined by the Predict Integral Copy Numbers In Cancer analysis [19], and have a total copy number of eight or more. Deletions must occur within a single contiguous segment with copy number zero. Wild type corresponds to a copy number range between 0 and 8, excluding both 0 and 8. Genes annotated as wild-type across all BC cell lines in terms of either CNV and mutation were removed. We also downloaded the dose-response curves for 266 drugs tested in 1065 cancer cell lines (including the 49 BC cell lines). Each curve is summarized by its IC_50_ value (potency).

RNA-Seq expression profiles of 28 BC cell lines, as well as their response measured by IC_50_ to 13 out of the GDSC drugs downloaded from CCLE (https://portals.broadinstitute.org/ccle) were used for model validation in this study. For the RNA-Seq data, we used expression level computed using the RNA-Seq by Expectation-Maximization (RSEM) method [20] provided by CCLE, multiplied by 10^6^ to obtain Transcripts Per Million (TPM) [20] and log2-transformed. Different drugs have different baseline values and ranges of response and in particular IC_50_ values can vary widely depending on experimental conditions. Therefore, for each of the two datasets (GDSC and CCLE), we subtracted the mean IC_50_ value (for all drugs) from each IC_50_ value and then divided this value by the standard deviation of IC_50_ values for all drugs. This normalization process gives different drugs the same baseline value and range across all BC cell lines for the GDSC and CCLE studies.

Drug chemical structure data were also curated. In order to reduce 3D drug chemical structure data into a 1D string variable we used the simplified molecular-input line-entry system (SMILES). We extracted the canonical SMILES strings for 220 out of the 266 GDSC small molecules from PubChem [21], a database of more than 60 million unique structures. We then used the *parse.smiles* function of the R package rcdk [22] to parse the annotated SMILES strings for existing drugs. Extended connectivity fingerprints (hash-based fingerprints, default length 1024) across all drugs were subsequently calculated using the *get.fingerprints* function of the R package rcdk. Regarding the drug targets, we extract the interactions between the 220 drugs and 272 target proteins from GDSC.

For the TCGA BC cohort, the patient-specific RNA-Seq gene expression data computed by the RSEM algorithm [20] were downloaded from Firehose Broad GDAC (https://gdac.broadinstitute.org), multiplied by 10^6^ to obtain TPM [20] and log2-transformed. We used clinical annotations of the drug response for some patients which were obtained from supplementary material of Ding et al.’s study [8]. We also used TCGA BC patients without drug response in their records. The data used in this study are summarized in Table 1.

**Table 1:** Data collected from multiple platforms

Dataset	Data type	Platform	Samples
GDSC	Gene expression	Affymetrix Human Genome U219 array	49 cell lines × 14,770 genes
	CNV	Affymetrix SNP6 array	49 cell lines × 3037 genes
	Mutation	Whole exome sequencing	49 cell lines × 8849 genes
	Drug response	IC_50_	49 cell lines × 220 drugs
CCLE	Gene expression	Illumina Hiseq 2000	28 cell lines × 14,770 genes
	Drug response	IC_50_	28 cell lines × 13 drugs
TCGA	Gene expression	Illumina Hiseq 2000	1100 tumors × 14,770 genes
	Drug response	RECIST response categories ^a^	110 tumors × 5 drugs
Drugs	Chemical structure	rcdk ^b^	220 drugs × 1024 fingerprints
	Target	Curated	220 drugs × 272 targets
MSigDB	Canonical pathways	Curated	1329 pathway gene sets

^a^ Response Evaluation Criteria in Solid Tumours (RECIST), a standard way to categorize treatment response of a cancer patient, including complete response, a partial response, progressive disease, and stable disease

^b^ An R package which can take the SMILES string of a drug as input and output the fingerprints, 1D- and 2D-structres of the drug

### Pathway activity score calculation

Gene sets of 1329 canonical pathways, which were curated from various pathway databases (e.g., KEGG, PID, REACTOME, and BIOCARTA), were extracted from the Molecular Signatures Database (MSigDB) website [23]. As described by Wang et al. [24]*,* we scored the pathway activities using the gene expression profiles of BC cell lines or patients from GDSC, CCLE or TCGA datasets. Note that we first standardized gene expression within each cohort, and then performed pairwise homogenization procedure before scoring pathway activities as described in other studies [7, 25] to make the expression measures in different datasets comparable. Briefly, we kept only genes presenting in all the three gene expression datasets (GDSC, CCLE and TCGA datasets) and applied the *ComBat* function from the sva R package [26] to adjust the potential batch effect in the data sets.

### Cell line similarity network construction

**Fig. 1:**
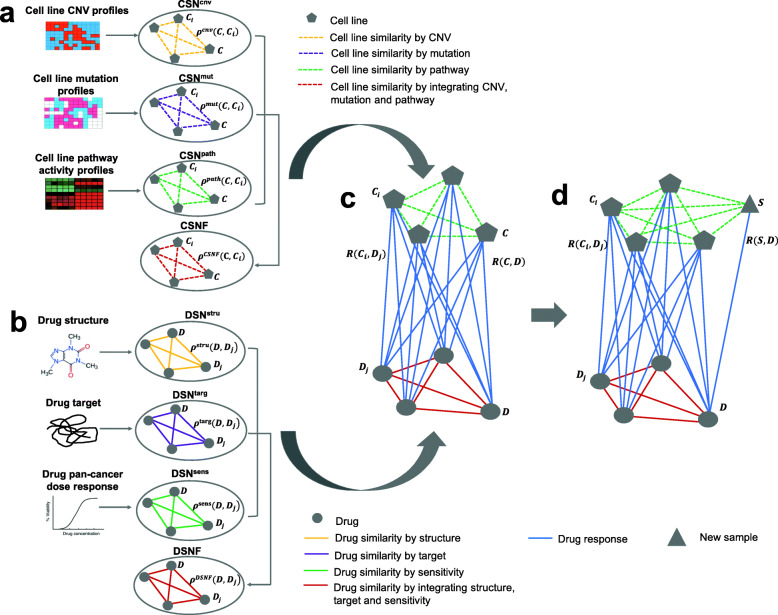
Study design. This study included four major steps: **a** four CSNs (CSN^cnv^, CSN^mut^, CSN^path^, CSNF) were constructed separately; **b** four DSNs (DSN^stru^, DSN^targ^, DSN^sens^, DSNF) were also constructed separately. **c** 16 ML-CDNs were constructed by connecting one of the four CDNs to one of the four DSNs, and here only ML-CDN2 was presented which was based CDN^path^ and DSNF. **d** ML-CDN2 was employed to predict the response of new breast cancer cell line samples from CCLE or new breast tumor tissue samples from TCGA to the existing drugs

We estimated the cell line similarities by constructing three CSNs using CNV, mutation, and pathway activity profiles (Fig. 1a): 1) CSN^cnv^, where associations between every two cell lines *C* and *C*_*i*_ are measured by the Tanimoto correlation (*ρ*^*cnv*^(*C*, *C*_*i*_)) between their CNV profiles; 2) CSN^mut^, which connects every cell line pair *C* and *C*_*i*_ using the Tanimoto correlation (*ρ*^*mut*^(*C*, *C*_*i*_)) calculated based on their gene mutation profiles; and 3) CSN^path^, which connects every two cell lines *C* and *C*_*i*_ based on their Pearson correlation (*ρ*^*path*^(*C*, *C*_*i*_)) of pathway activity profiles. Hence, these CNSs are weighted networks. The CSNs were generated using GDSC data and all of them are complete graphs of 49 BC cell lines, where the weights of the interactions between each pair of the cell lines were measured by the correlation coefficients of their respective pathway activity, CNV, and mutation profiles. Each of the three CSNs is a single-layer CSN since each was constructed using a single data type. Next, we used the Similarity Network Fusion (SNF) algorithm in the R SNFtool package [27] to integrate the three CSNs in two steps: 1) an affinity matrix was calculated from each CSN using the *affinityMatrix* function with default parameters; 2) the three affinity matrices were fused into a cell line similarity network fusion (CSNF) using the *SNF* function, which connects every cell line pair *C* and *C*_*i*_ by the SNF algorithm-derived correlation (*ρ*^*CSNF*^(*C*, *C*_*i*_)). The CSNF is a three-layer CSN since it combined CSN^path^, CSN^cnv^, and CSN^mut^.

### Drug similarity network construction

Three drug data types (drug structure, target, and pan-cancer response information) were used to construct three DSNs to estimate drug similarities (Fig. 1b): 1) DSN^stru^, which connects every two drugs *D* and *D*_*j*_ based on the Tanimoto correlation (*ρ*^*stru*^(*D*, *D*_*j*_)) of their molecular fingerprint properties; 2) DSN^targ^, where associations between every two drugs *D* and *D*_*j*_ are measured by the Jaccard correlation (*ρ*^*targ*^(*D*, *D*_*j*_)) between their target information; and 3) DSN^sens^, which connects every drug pair *D* and *D*_*j*_ using the Pearson correlation (*ρ*^*sens*^(*D*, *D*_*j*_)) calculated based on their sensitivity profiles with respect to the IC_50_ values across the 1065 cell lines from all cancer types. Next, we integrated the three single-layer DSNs into a three-layer drug similarity network fusion (DSNF) using the SNFtool package [27] by connecting every drug pair *D* and *D*_*j*_ using the SNF correlation (*ρ*^*DSNF*^(*D*, *D*_*j*_))*.*

### Multiple-layer cell line-drug response network prediction construction

We next integrated each of the four CSNs and each of the four DSNs together to develop a multiple-layer cell line-drug response network (ML-CDNs), which predicts drug response in previously tested BC cell lines (Fig. 1c). In this ML-CDN model, a layer denotes a data type from either the cell lines or the drugs. A ML-CDN connects a CSN and a DSN by linking the cell lines in the first network to their corresponding (previously tested) drugs in the second network. A ML-CDN is a bipartite graph of all cell lines and drugs, labeled with the corresponding response values (IC_50_ values). Note that a ML-CDN is not a complete bipartite graph due to some missing values in the GDSC dataset. With the four CSNs and four DSNs constructed in the above two sections, we obtained a total of 16 ML-CDNs: 1) nine dual-layer CDNs were built using each of the three single-layer CSNs (CSN^path^, CSN^cnv^ and CSN^mut^) and each of the three single-layer DSNs (DSN^stru^, DSN^targ^, DSN^sens^); 2) three four-layer CDNs were built using the three-layer CSNF combined with each of the three single-layer DSNs; 3) similarly, another three four-layer CDNs were built using the three-layer DSNF combined with each of the three single-layer CSNs; 4) finally, a six-layer CDN was built using the two integrated similarity networks, CSNF and DSNF. In this study, we referred to the ML-CDN based on CSNF and DSNF as ML-CDN1 and the one based on CSN^path^ and DSNF as ML-CDN2.

For a given cell line-drug pair (*C*, *D*), we are able to make a prediction of the response of the cell line C to the drug D using Eq. 1, where Ω is the set of all possible cell line-drug pairs, *Ω*\{(*C*, *D*)} is the set of all other pairs (*C*_*i*_, *D*_*j*_) except (*C, D*), *R*(*C*_*i*_, *D*_*j*_) denotes the observed response of the pair (*C*_*i*_, *D*_*j*_). $\hat{R}\left(C,D\right)$ is the predicted response value for the pair (*C*, *D*). The product of *w*(*C*, *C*_*i*_) and *w*(*D*, *D*_*j*_) reflects the contribution of *R*(*C*_*i*_, *D*_*j*_) to $\hat{R}\left(C,D\right)$.

1 $$ \hat{R}\left(C,D\right)=\frac{\sum_{\left({C}_i,{D}_j\right)\in \varOmega \backslash \left\{\left(C,D\right)\right\}}w\left(C,{C}_i\right)w\left(D,{D}_j\right)R\left({C}_i,{D}_j\right)}{\sum_{\left({C}_i,{D}_j\right)\in \varOmega \operatorname{}\left\{\left(C,D\right)\right\}}w\left(C,{C}_i\right)w\left(D,{D}_j\right)} $$

where *w*(*C*, *C*_*i*_) is the weight function between cell lines *C* and *C*_*i*_ and *w*(*D*, *D*_*j*_) is the weight function between drugs *D* and *D*_*j*_, which can be calculated as Eqs. 2 and 3. The weight *w*(*C*, *C*_*i*_) increases with respect to the cell line similarity correlation *ρ*(*C*, *C*_*i*_), and *σ* measures the decay rate when *ρ*(*C*, *C*_*i*_) decreases. Similarly, the parameter *τ* measures the decay rate with the decrease of *ρ*(*D*, *D*_*j*_).

2 $$ w\left(C,{C}_i\right)={e}^{-\frac{{\left[1-\rho \left(C,{C}_i\right)\right]}^2}{2{\sigma}^2}} $$

3 $$ w\left(D,{D}_j\right)={e}^{-\frac{{\left[1-\rho \left(D,{D}_j\right)\right]}^2}{2{\tau}^2}} $$

The ML-CDN models contain two decay parameters (*σ*, *τ*), to be used in the weight function of cell lines and drugs, respectively. The decay parameter pair (*σ*, *τ*) was optimized by minimizing the sum of the squared errors for all possible cell line-drug pairs using Eq. 4 as the response prediction model. In detail, the overall error function is defined as Eq. 4, where *R*(*C*, *D*) is the observed response value of cell line *C* to drug *D*, and $\hat{R}\left(C,D\right)$ is the predicted value of the cell line *C* to the drug *D*. *σ* and *τ* are both ranged from 0 to 1 with an increment of 0.01, and the pair (*σ*, *τ*) takes all possible combinations.

4 $$ J\left(\sigma, \tau \right)={\sum}_{\left(C,D\right)\in \Omega}{\left(R\left(C,D\right)-\hat{R}\left(C,D\right)\right)}^2 $$

To compare the performance of the 16 ML-CDN models, we split all cell line-drug pairs into three folds. Two folds were used as the training set for optimizing the decay parameter pairs (*σ*, *τ*) while the remaining fold used as the test set for estimating the prediction performance of the models. The performance was evaluated using Pearson correlation coefficient and root mean squared error (RMSE) between the predicted and the observed drug responses for all drugs. RMSE is the square root of the mean squared error. A higher Pearson correlation coefficient and lower RMSE indicate a better prediction performance of a method.

### Multiple-layer cell line-drug response network model to predict drug response for a new sample

Although the ML-CDN models can be used to predict drug response in a new cell line or tumor tissue sample based on the CSN, or cell line response to a new drug based on the DSN, we focused on the former in this study. We therefore expected that the BC cell line-derived ML-CDNs could have good predictive performance in BC patient-derived data. Among the 16 ML-CDN models, ML-CDN2, which was constructed by connecting CSN^path^ and DSNF and demonstrated good prediction performance in terms of Pearson correlation and RMSE (See the Results Section), was chosen for predicting the response of a new BC patient- or cell line-derived sample *S* to a known drug *D* ($\hat{R}\left(S,D\right)\Big)$ (Fig. 1d) as defined in Eq. 5. To make a prediction for *R*(*S*, *D*), we first estimated the similarity between the new sample *S* and any existing cell line *C*_*i*_ by calculating the Pearson correlation (*ρ*^*path*^(*S*, *C*_*i*_)) in terms of their pathway activity profiles and further obtained the weight (*w*^*path*^(*S*, *C*_*i*_)) between *S* and *C*_*i*_ using Eq. 2 with *σ* optimized from ML-CDN2. In parallel, the similarity between *D* and any existing drugs *D*_*j*_ was measured using *ρ*^*DSNF*^(*D*, *D*_*j*_) from DSNF and further weighted using Eq. 3 with *τ* optimized from ML-CDN2 to obtain *w*^*DSFN*^(*D*, *D*_*i*_). *R*(*C*_*i*_, *D*_*j*_) is the observed response value of existing cell line *C*_*i*_ to existing drug *D*_*j*_. Thus, *R*(*S*, *D*) can be predicted by taking advantage of response data from all existing cell lines *C*_*i*_ based on their weighted similarities with the new sample *S* and all existing drugs *D*_*j*_ based their weighted similarities with *D* as shown in Eq. 5. The predictions of the response to the existing drugs in the benchmark dataset (i.e., GDSC) were made for new BC cell lines from CCLE or new breast tumor tissue samples from TCGA.

5 $$ \hat{R}\left(S,D\right)=\frac{\sum_{\left({C}_i,{D}_j\right)\in \varOmega }{w}^{path}\left(S,{C}_i\right)\ {w}^{DSFN}\left(D,{D}_j\right)\ R\left({C}_i,{D}_j\right)}{\sum_{\left({C}_i,{D}_j\right)\in \varOmega }{w}^{path}\left(S,{C}_i\right)\ {w}^{DSFN}\left(D,{D}_j\right)} $$

### Drug response prediction for CCLE cell lines

To validate the performance of ML-CDN2, we employed the ML-CDN2 model, which was trained using the GDSC dataset. We then predicted the drug response of the same drugs in new BC cell lines from the CCLE dataset using Eq. 5. First, we measured the similarities between new cell lines from CCLE and the existing cell lines from GDSC using the Pearson correlations of their pathway activity profiles and then obtained the weights between the CCLE and GDSC BC cell lines using Eq. 2 with *σ* optimized from ML-CDN2. The prediction of the drug response could then be made using Eq. 5.

### Drug response prediction for TCGA patients

To further study ML-CDN2’s performance in vivo, we employed the model to predict the drug response of five drugs for patients in the TCGA BC dataset for which drug response was recorded. Five drugs were tested in the GDSC study, including paclitaxel, fluorouracil, tamoxifen, doxorubicin, and docetaxel. For each of the five drugs, the patients were assigned to two groups based on the recorded drug response: Responder (patients showing a “complete response”) and Non-responder (patients showing a “partial response”, “progressive disease”, or “stable disease”). For these patients, we first calculated their pathway activity scores based on their whole-genome gene expression profiles and then measured the similarity between these BC tumors and the GDSC BC cell lines using the Pearson correlations of their pathway activity profiles. The Pearson correlations were further weighted using Eq. 2 with *σ* optimized from ML-CDN2. In the end, the responses of these TCGA BC patients to the five drugs were made with Eq. 5. Since the IC_50_ values in the GDSC study were measured using cell viability, we expected that patients in the Responder group would have a lower predicted IC_50_ value than patients in the Non-responder group.

Using Eq. 5, we also predicted the response of all TCGA BC patients to lapatinib, and tamoxifen, which were included in the GDSC study. Lapatinib is a tyrosine kinase inhibitor targeting HER2/EGFR receptors and is used to treat HER2-overexpressing breast cancers. Tamoxifen is a selective estrogen receptor modulator (SERM) that targets ER receptors and is used to treat ER-positive breast cancers. For lapatinib, we separated the BC patients based on their HER2 overexpression level measured by immunohistochemistry (IHC) into four groups: 0, 1+, 2+, 3+, indicating the increasing expression level of HER2. We then compared the predicted IC_50_ values for lapatinib among the four groups. We expected to see that groups with higher HER2 expression levels would demonstrate lower predicted IC_50_ values. BC patients treated with tamoxifen were divided into two groups (Negative and Positive) based on the IHC status of ER. Then the predicted IC_50_ values for tamoxifen were compared between the two groups. We expected that the predicted IC_50_ values for patients in the ER Positive group would be lower than the IC_50_ values from the patients in the ER Negative group.

In addition, we used Eq. 5 to predict the drug response of the EGFR and PI3K pathway inhibitors for TCGA BC patients for which there was no drug response recorded. Since these drugs target either the EGFR pathway or the PI3K pathway, we expected the expression level of the EGFR pathway genes to be strongly correlated with the predicted EGFR inhibitor response while the expression level of the PI3K pathway genes would be strongly correlated with the predicted PI3K inhibitor response. We obtain the gene lists for the EGFR and PI3K pathways from MSigDB [23]. To study the correlation between genes in a pathway and an inhibitor of the pathway, we employed multiple linear regression between the predicted IC_50_ value (response variable) of the inhibitor and the expression levels of the pathway genes (predictors). We obtained the *p*-value for each gene and corrected them for multiple comparison, using Bonferroni correction (*α* = 0.05).

## Results

### The cell line and drug data types are associated with drug response

**Fig. 2:**
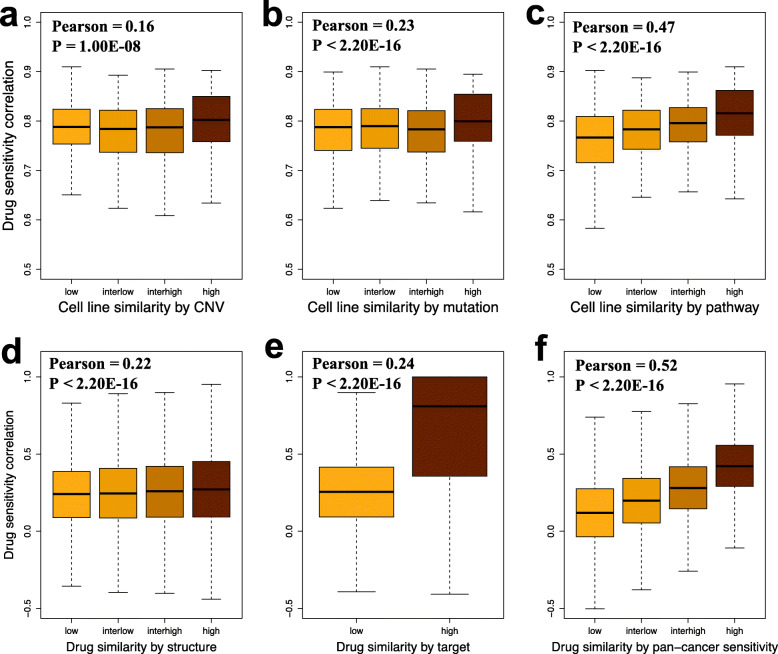
Similar cell lines and similar drugs have similar responses. **a**, **b**, and **c** Box plots show that cell lines with similar CNV (**a**), mutation (**b**), and pathway activity (**c**) profiles respond similarly to the same drugs. The X-axis indicates the similarity level between all possible BC cell line pairs. We divided the similarity level between two cell lines into “low” (minimum <= *ρ*(*C*, *C*_*i*_) < 1st quantile), “interlow” (1st quantile <= *ρ*(*C*, *C*_*i*_) < median), “interhigh” (median < = *ρ*(*C*, *C*_*i*_) < 3rd quantile) and “high” (3rd quantile <= cc < maximum) based on CNV (**a**), mutation (**b**), and pathway activity (**c**) correlations. The y-axis shows the correlations of their drug response vectors as measured by IC_50_. **d**, **c**, **e** Box plots show that drugs with similar structure (**d**), targets (**e**), and pan-cancer cell line sensitivity (**f**) profiles have similar effects on BC cell lines. The drug pairs were separated into “low” (minimum <= *ρ*(*D*, *D*_*j*_) < 1st quantile), “interlow” (1st quantile <= *ρ*(*D*, *D*_*j*_) < median), “interhigh” (median < = *ρ*(*D*, *D*_*j*_) < 3rd quantile) and “high” (3rd quantile <= *ρ*(*D*, *D*_*j*_) < maximum) groups based on the correlations of structure (**a**) and pan-cancer cell line sensitivity (**c**) profiles. Drug pairs were divided into “low” (*ρ*^*targ*^(*D*, *D*_*j*_) <= 0) and “high” (*ρ*^*targ*^(*D*, *D*_*j*_) > 0) groups using their target correlations. The Y-axis shows the correlations of their sensitivity vectors tested in BC cell lines as measured by IC_50_

To measure the similarity of cell line pairs, we calculated the correlations between pairs of cell lines using their CNV, mutation, and pathway activity profiles (Additional file 1: Table S1). The mean correlation of all cell line pairs is 88.12% for CNV, 88.10% for mutation and 96.83% for pathway activity. The Pearson correlation between the drug response similarity and the cell line pair similarity is 0.16 for CNV, 0.23 for mutation, and 0.47 for pathway activity (Fig. 2). Figure 2 shows that if two BC cell lines show similar patterns in terms of the CNV (Fig. 2a), mutation (Fig. 2b), and pathway activity (Fig. 2c) profiles, their responses to certain drugs will be similar. We also found that drug pairs with similar structures (Fig. 2d), targets (Fig. 2e), and pan-cancer IC_50_ profiles (Fig. 2f) exhibit similar IC_50_ values across the BC cell lines tested. For example, the mammary gland cell lines BT-20 and HCC1187 have a correlation of 0.99 for their CNV profiles, and their correlation in terms of their response to all tested drugs in GDSC is 0.81 (Additional file 1: Table S1). These results suggest that integrating cell line and drug data types may improve drug response prediction.

### Comparison of ML-CDN models

The three types of cell line information enumerated, and the three types of drug information enumerated in Fig. 2 are associated with drug response. We therefore used each of the four CSNs (CSN^cnv^, CSN^mut^, CSN^path^, and CSNF) and each of the four DSNs (DSN^stru^, DSN^targ^, DSN^sens^, and DSNF) to build a total of 16 ML-CDNs and compared their prediction performance (Table 2). The optimal model is ML-CDN1 which was constructed with CSNF and DSNF. Among the other models with high predictive capabilities, we focused on ML-CDN2 which was constructed from CSN^path^ and DSNF. This is because pathway activity profiles derived from the transcriptome are the most widely available data for tumor tissue or cell line samples in public databases and the DSNF integrated three types of information from drugs.

**Table 2:** Performance of the 16 ML-CDN models

CSN	DSN	RMSE	R
CSN^cnv^	DSN^stru^	0.513	0.864
CSN^cnv^	DSN^targ^	0.562	0.834
CSN^cnv^	DSN^sens^	0.504	0.869
CSN^cnv^	DSNF	0.504	0.869
CSN^mut^	DSN^stru^	0.513	0.864
CSN^mut^	DSN^targ^	0.562	0.834
CSN^mut^	DSN^sens^	0.505	0.869
CSN^mut^	DSNF	0.504	0.869
CSN^path^	DSN^stru^	0.510	0.866
CSN^path^	DSN^targ^	0.563	0.833
**CSN**^**path**^	**DSN**^**sens**^	**0.496**	**0.873**
**CSN**^**path**^	**DSNF**	**0.497**	**0.873**
CSNF	DSN^stru^	0.511	0.865
CSNF	DSN^targ^	0.561	0.834
**CSNF**	**DSN**^**sens**^	**0.496**	**0.873**
**CSNF**	**DSNF**	**0.493**	**0.875**

*R* Pearson correlation coefficient, *RMSE* Root mean squared error

**Fig. 3:**
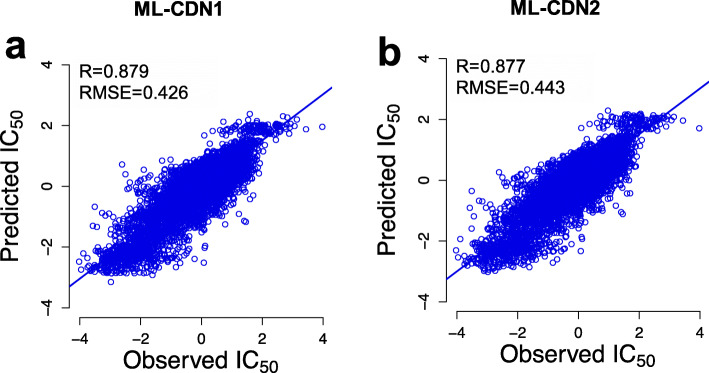
Comparison of ML-CDN1 and ML-CDN2. **a** The scatter plot of observed and predicted drug responses (IC_50_ values) for all drugs in GDSC using the ML-CDN1 model. **b** The scatter plot of observed and predicted drug responses (IC_50_ values) for all drugs in GDSC using the ML-CDN2 model. R: Pearson correlation coefficient; RMSE: root mean squared error

We optimized the decay parameters for ML-CDN1 and ML-CDN2 models using all cell line-drug pairs. The optimized parameter pair (*σ*, *τ*) is (0.07, 0.06) for ML-CDN1 and (0.01, 0.06) for ML-CDN2. The IC_50_ values were predicted for all pairs using the ML-CDN1 and ML-CDN2 models to calculate the Pearson correlation and RMSE. The scatter plots for ML-CDN1 (Fig. 3a**)** and ML-CDN2 (Fig. 3b) indicate good correlations between observed versus predicted responses which did not arise from a small number of outliers. We decided to use ML-CDN2 to predict drug response for new cell line-derived from tumor tissue samples because the two models do not differ much in their performance and ML-CDN2 does not use CNV and mutation data whereas the ML-CDN1 model does.

### Comparing ML-CDN2 with other methods

**Fig. 4:**
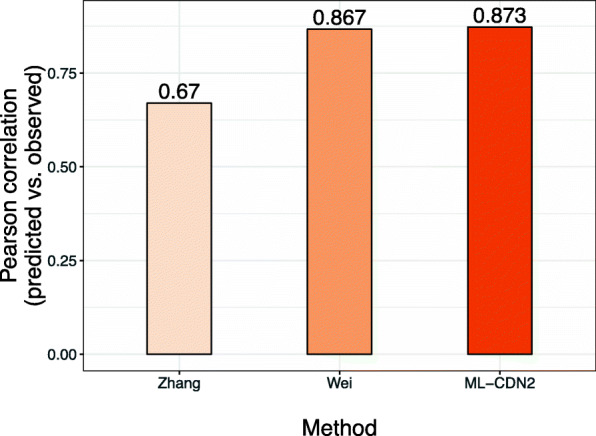
Comparison of ML-CDN2 and other network-based models. The bar graph shows the predictive performance of three models, which was estimated based on Pearson correlations (the number on the top of bars) between the predicted and observed IC_50_ values. The first (Zhang) is Zhang et al.’s method. The second (Wei) is Wei et al.’s method. The third is the ML-CDN2 model from in this study using the CSN^path^ and DSNF

We compared the performance of ML-CDN2 with a dual-layer network proposed by Zhang et al. [14]. Our ML-CDN2 model is similar to the method of Zhang because both use CSN and DSN to predict the drug response for a given cell line-drug pair but it differs because Zhang et al constructed a dual-layer integrated cell line-drug network model by combining the predictions from the individual layers. We used the same 220 drugs and 49 BC cell lines from the GDSC study for evaluation in order to make fair comparisons. Following the method of Zhang et al., the CSN was generated using the cell line pairwise Pearson correlations of their gene expression profiles. We extracted the 1-D and 2-D structural features of each drug using PaDEL [28] and calculated the Pearson correlation between each pair of the drugs using these structural features to build the DSN. Our ML-CDN2 performed better than Zhang et al.’s method (Fig. 4**)**. The ML-CDN2 model obtained a correlation between the predicted and the observed responses of 0.873, while Zhang et al.’s method had a value of 0.670 (Fig. 4).

We also compared our method with that of Wei et al. [15], which predicts anticancer drug response by capturing the different contributions of existing cell line-drug responses through cell line similarities and drug similarities. Our ML-CDN2 method uses the same strategy to integrate the CSN and DSN. However, Wei et al.’s method measured cell line similarity using only gene expression profiles and the drug similarity using only fingerprint-based chemical structures. Our ML-CDN2 performed better than the model of Wei et al. (Fig. 4).

### Predicting missing drug responses in GDSC

**Fig. 5:**
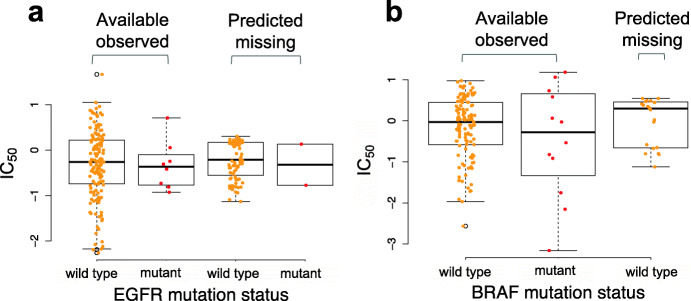
Comparison of the predicted missing response values using ML-CDN2 and the existed response values for two types of inhibitors. **a** Comparison of predicted missing and available observed IC_50_ values for EGFR mutant and wild-type cell lines for which experimental IC_50_ values were missing from the GDSC dataset for EGFR inhibitors, including afatinib, cetuximab, erlotinib, gefitinib and lapatinib. **b** Comparison of predicted missing and available observed IC_50_ values for BRAF mutant and wildtype cell lines for which experimental IC_50_ values were missing in the GDSC dataset for three MEK inhibitors, including AZD6244, RDEA119 and PD-0325901

Out of the possible 49 × 220 BC cell line-drug combinations in the GDSC study, only 81% have corresponding drug response data. With the cell line similarity and drug similarity data, we applied our ML-CDN2 model to predict the missing IC_50_ values for these pairs without responses (predicted missing) and compared this to those with responses (available observed). We predicted the missing responses to five EGFR tyrosine kinase inhibitors (afatinib, erlotinib, gefitinib and lapatinib). Since such EGFR inhibitors are more potent (lower IC_50_) in individuals with mutations in EGFR, we stratified the data into wild type and mutant groups. The predicted missing median IC_50_ values of EGFR inhibitors for the EGFR wild type and mutant cell lines are − 0.09 and − 0.32, respectively, and the available observed median IC_50_ values for the EGFR wild type and mutant groups are 0.10 and − 0.28, respectively (Fig. 5a). Although the wild-type median EGFR IC_50_ values appear to be consistently higher than the mutant median EGFR IC_50_ values, these differences are not significant within the available observed, and predicted missing groups. For example, the predicted missing wild type and mutant IC_50_ values are not significantly different (*p*-value = 0.73, two-tailed t-test), likely because there are only two data points in the mutant group. However, we found that the predicted missing median IC_50_ of the EGFR-wild type group was significantly higher than the available observed median IC_50_ of the EGFR-mutant cell lines (*p*-value = 0.05 two-tailed t-test) (Fig. 5a). Our findings agree with previously published studies [15, 29]. Moreover, these results are consistent with the fact that EGFR tyrosine kinase inhibitors usually only work in individuals with activating mutations in the EGFR tyrosine kinase domain which makes the drugs have lower IC_50_ values in these individuals.

Similar to the EGFR inhibitors, we predicted the missing responses of three mitogen-activated protein kinase inhibitors (AZD6244, RDEA119 and PD-0325901). These compounds are expected to be more potent and therefore have lower IC_50_ values in cells that harbour a specific mutation in the BRAF kinase [30], so we stratified the data into wild type and mutant groups. We found that all of the cell lines with missing responses to the three inhibitors were BRAF wild type, so we could not predict missing BRAF mutant values. The predicted missing median IC_50_ value in the BRAF-wild type cell lines using ML-CDN2 is 0.29 which is close to the available observed median IC_50_ of − 0.03, and both values are higher than the available observed median IC_50_ of − 0.28 in the BRAF-mutant cell lines (Fig. 5b). However, we did not find a significant difference between the predicted missing median IC_50_ value in BRAF-wild type cell lines and the available observed IC_50_ value in the BRAF-mutant cell lines (*p*-value = 0.3, two-tailed t-test). Overall, our results suggest that our ML-CDN2 model can correctly predict the drug responses of missing data in the GDSC dataset.

### Validating ML-CDN2 in CCLE

**Fig. 6:**
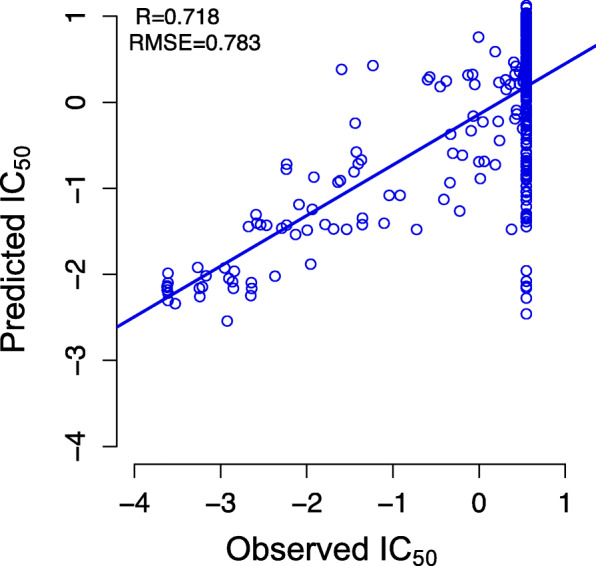
Correlation between the predicted and observed drug responses using ML-CDN2 in CCLE. Many cell lines have an observed IC_50_ of 0.55 corresponding to the maximum dose of 8 μM in the CCLE IC_50_ curves. The true value of the IC_50_ for these points is 8 **μ**M or higher. However, the IC_50_ values predicted by ML-CDN2 were not subject to this limitation. Therefore, we cannot see a good correlation between the predicted and observed IC_50_ values for the pairs that had an observed IC_50_ value of 8 **μ**M or more

We next validated ML-CDN2 in the CCLE dataset using 13 drugs tested in both CCLE and GDSC and 28 BC cell lines with gene expression data available. Treating each cell line as a new sample, we employed ML-CDN2 to predict the responses of the new sample to the 13 drugs. The Pearson correlation coefficient between the observed and predicted drug responses is 0.718 with a RMSE of 0.783 (Fig. 6**)**. The results suggest that the ML-CDN2 model can be used to predict response values of new BC cell lines to the existing drugs. However, the model did not work well with cell-drug pairs with observed IC_50_ values of 8 μM or higher. In CCLE, drugs were tested in eight doses with 8 μM being the maximum. Thus, some drugs ended up having an IC_50_ of 8 μM or higher in some cell lines but these are all listed as having an IC_50_ of 8 μM. The IC_50_ of 8 μM is 0.55 after normalization, hence all IC_50_ values of 8 μM or higher from CCLE are represented as the observed normalized value of 0.55 (Fig. 6). Unfortunately, we cannot exclude the 8 μM IC_50_ values because we don’t know which IC_50_ values are legitimately 8 μM and which are higher. Therefore, some of the predicted IC_50_ values cannot be linearly correlated with the normalized observed IC_50_ values of 0.55 because of a limitation in the data from CCLE.

### Evaluating ML-CDN2 in TCGA

**Fig. 7:**
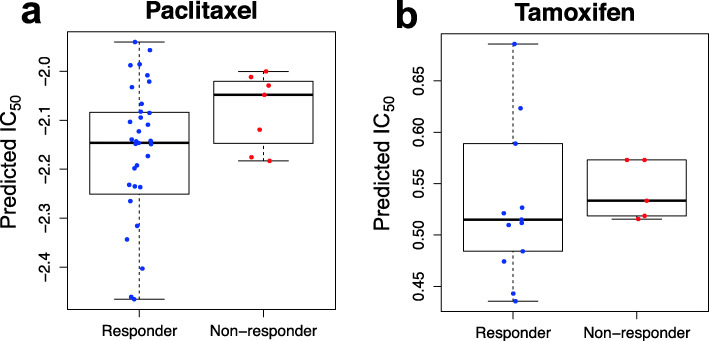
Predicted IC_50_ values for TCGA BC samples with recorded drug response. **a** The boxplot shows the predicted IC_50_ values for TCGA responders and non-responders to paclitaxel treatment. **b** The boxplot shows the predicted IC_50_ values for TCGA responders and non-responders to tamoxifen treatment

Although drug response data in BC cell lines is more widely available and easier to obtain, it is less representative of the disease than human tumor samples. In addition, a goal of models such as ours is to predict successful cancer drug treatment based on BC patient tumor samples. We therefore tested our ML-CDN2 against the TCGA dataset on a subset of BC patients where their drug response was recorded as “Responder” or “Non-responder”. The predicted drug IC_50_ values were lower in the “Responder” group to paclitaxel treatment, compared to the “Non-responder” group (Fig. 7a, *p*-value = 0.01, two-tailed t-test). In the tamoxifen treatment group, patients in the “Responder” group appeared to have a lower median predicted IC_50_ value than patients in the “Non-responder” group but the results are not statistically significant (Fig. 7b, *p*-value = 0.93, two-tailed t-test).

**Fig. 8:**
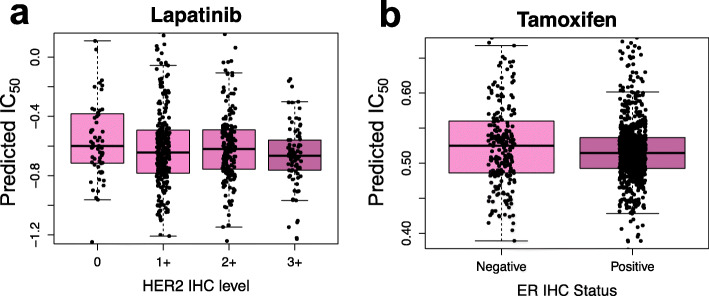
Predicted IC_50_ values for TCGA BC samples without recorded drug response. **a** The boxplot shows the predicted lapatinib response values in TCGA BC groups with different HER2 overexpression level, which was measured by IHC. **b** The boxplot shows the predicted tamoxifen response values in TCGA BC groups with different ER expression status, which was also measured by IHC

ML-CDN2 was used to predict the response of all TCGA BC samples to lapatinib, a HER2/EGFR tyrosine kinase inhibitor used to treat HER2 positive BC, and tamoxifen, a SERM used to treat ER positive BC. When grouping the TCGA BC patients based on their HER2 IHC status. We found that the predicted responses among the four groups were significantly different (Fig. 8a, *p*-value = 0.01 by ANOVA). Tamoxifen responses were similarly grouped by ER status. Although tamoxifen appeared to be more sensitive in the ER-positive than in the ER-negative group this result was not statistically significant (Fig. 8b). These results further suggest that the GDSC BC cell line-derived ML-CDN2 could be used to predict the responses of BC patients to existing drugs.

### Drug response predictions for TCGA samples have significant associations with expression levels of targeted pathway genes

We applied the ML-CDN2 model to the gene expression data from TCGA BC samples that did not have drug response data and predicted the response to seven drugs targeting the EGFR signaling pathway: erlotinib, gefitinib, afatinib, cetuximab, lapatinib, CP724714, and pelitinib. A number of strong associations between the EGFR pathway genes and the responses to the six drugs were predicted by ML-CDN2 (Table 3). For example, for erlotinib, we observed statistically significant associations between the expression of EGFR pathway genes *ADCY7*, *CDK1*, *FOXO1*, *MAPK1*, and *PAG1* and the predicted responses. However, no such significant associations were observed with gefitinib.

**Table 3:** Associations between the expression level of EGFR pathway genes and EGFR inhibitor responses predicted by ML-CDN2

Drug	Gene	Adjusted ***p***-value
Erlotinib	*ADCY7*	1.51 × 10^−3^
	*CDK1*	2.00 × 10^−31^
	*FOXO1*	4.22 × 10^−2^
	*MAPK1*	1.02 × 10^−2^
	*PAG1*	2.64 × 10^−3^
Afatinib	*ADAM12*	1.64 × 10^−5^
	*ADCY7*	4.63 × 10^−4^
	*CDK1*	8.88 × 10^− 3^
	*EGF*	2.94 × 10^− 2^
Cetuximab	*ADCY6*	2.67 × 10^− 2^
	*ADRBK1*	1.02 × 10^− 2^
	*CDK1*	1.59 × 10^−19^
	*SPRY2*	2.18 × 10^− 4^
Lapatinib	*AKT3*	8.00 × 10^−3^
	*CDK1*	5.68 × 10^−17^
	*ITPR2*	2.39 × 10^−5^
CP724714	*CDK1*	6.22 × 10^−9^
	*FOXO1*	3.18 × 10^−2^
	*PDPK1*	2.17 × 10^−2^
Pelitinib	*ADCY9*	4.06 × 10^−4^
	*CDK1*	9.07 × 10^−5^
	*EGFR*	2.90 × 10^−2^
	*ITPR3*	3.03 × 10^−3^
	*PAG1*	3.87 × 10^−2^
	*PIK3R1*	1.04 × 10^−2^

We also employed the ML-CDN2 model to predict the drug response of several phosphoinositide 3-kinase (PI3K) / mechanistic target of rapamycin (mTOR) signaling pathway inhibitors for TCGA breast tumor samples. 20 PI3K inhibitors were tested in the GDSC study and we observed statistically significant associations between the level of pathway gene expression and predicted drug responses for each inhibitor. A total of 120 associations were obtained for seven inhibitors (Additional file 2: Table S2). For example, we observed significant associations between the predicted responses to dactolisib and the expression of the genes *CXCR4* (*p*-value = 2.16 × 10^− 3^) and *FASLG* (*p*-value = 2.60 × 10^− 2^).

## Discussion

All of the 16 ML-CDN models we constructed show good predictive performance, with Pearson correlation coefficients greater than 0.8. When the same CSN was used, the ML-CDN model derived from DSN^targ^ shows a smaller Pearson correlation while having a greater RMSE than the ML-CDN models derived from DSN^stru^, DSN^sens^, and DSNF. These findings suggest that the drug targets are less predictive of drug response in the ML-CDN models compared to the drug structures, pan-cancer IC_50_ profiles, and DSNF. When the same DSN (except DSN^targ^) was used, the ML-CDN models derived from CSN^path^ and CSNF show a higher Pearson correlation and a lower RMSE than the ML-CDN models derived from CSN^cnv^ and CSN^mut^, implying that pathway activity profiles and CSNF are more informative than the CNV and mutation profiles. The best-preforming model is ML-CDN1, derived from CSNF and DSNF, suggesting that integration of the three types of data from cell lines along with the three types of data from drugs improve predictive performance of the model.

It is noteworthy that the 16 ML-CDNs do not differ much in their predictive performance. The Pearson correlation ranges from 0.833 to 0.875 while the RMSE ranges from 0.493 to 0.563. A possible reason for this could be that the ML-CDN models are BC-specific models. The idea behind the ML-CDN modeling method is that similar cell lines exhibit similar drug response. These models were trained on 49 BC cell lines, which show high similarity among each other in terms of pathway activity, CNV, or gene mutation profiles. Therefore, any of the three types of profiles can be used by the ML-CDN model to accurately predict drug response.

When we tested our ML-CDN2 against the TCGA dataset, the model predicted significantly lower IC_50_ values for the paclitaxel “Responders” than the “Non-responders” (Fig. 7a). Our model did not capture variability in clinical response to the other four drugs. Notably, for tamoxifen, patients in the “Responder” group had a lower median predicted IC_50_ value than patients in the “Non-responder” group (Fig. 7b). However, given that there were only five individuals in the Non-responder group, it is not surprising that we did not establish statistical significance. Consequently, a larger clinical cohort may be required to assess rigorously whether our models capture variability in tamoxifen response for BC.

Recent studies have demonstrated that computational models built on cell line-derived data are applicable to the prediction of drug response for cancer patient samples [7, 11, 31]. For example, Geeleher et al. [7] developed ridge regression models for single drugs using the baseline gene expression data of cancer cell lines as input and the in vitro drug IC_50_ values as output. Geeleher et al. [31] also applied this ridge regression model to the gene expression profiles of the TCGA tumors to determine drug response and showed that their cell line-derived models can be used for the accurate prediction of drug response in TCGA tumor samples. In our study, the ML-CDN2 model, which was developed on the GDSC dataset, has demonstrated the potential to predict anti-cancer drug response for breast tumor tissue samples from TCGA. These findings suggest that cell-line derived CNV, gene expression, and mutation data can be used for developing computational drug response prediction models, which could be applied to precision medicine *.*

Gene expression data have been extensively used both as a single input and in combination with other omics data for in silico drug response prediction [6]. However, most of these models focused on the expression of individual genes. Recent evidence has shown that drug responses are mediated by the coordinated function of a set of genes (i.e., a pathway) instead of individual genes [32]. In this study, we inferred the pathway activity profiles from the gene expression data. We estimated the similarity between two GDSC cell lines by calculating the Pearson correlation of their pathway activity profiles, resulting in a mean correlation of all possible cell line pairs of 97.49%, which is higher than the mean (96.83%) of cell line pairwise correlations of the gene expression profiles (Additional file 3: Fig. S1). We also estimated the similarity between pairs of TCGA BC tumors by computing the Pearson correlation with respect to their gene expression profiles as well as their pathway activity profiles. The mean correlation of all BC tumors pairs is 80.33% for gene expression and 97.49% for pathway activity (Additional file 3: Fig. S1). Moreover, we measured the similarity between the GDSC BC cell lines and the TCGA breast tumor samples. The mean correlation of all possible cell line-tumor pairs is 88.20% for gene expression and 96.57% for pathway activity (Additional file 3: Fig. S1). These findings suggest that pathway activity profiles provide a better way to estimate the similarity of cancer cell line pairs, tumor pairs, as well as cell line-tumor pairs, than the expression profiles of individual genes.

A limitation of this study was that our model did not take into account cell lines that are known to be resistant to specific chemotherapeutic drugs. Therefore, when we validated our model in the CCLE dataset, it did not work well with cell-drug pairs with observed IC_50_ values of 8 μM or higher. Triple-negative breast cancer (TNBC) is the most aggressive BC subtype with the lowest survival time and having few effective therapies. Our model was developed with different BC subtypes taken into consideration and was validated using different BC subtypes represented by cell lines in CCLE and patient-derived samples in TCGA. It would be interesting to look at the performance of our model specifically in the TNBC subtype in future work.

## Conclusions

We developed a BC-specific computational model by integrating multiple cell line and drug data types to predict anticancer drug responses. One of the main contributions is that this BC cell line-derived model has the potential to predict the drug response for BC tissue samples. Such a model may one day be used to predict drug response and influence drug selection for BC treatment.

## Supplementary Information

**Additional file 1: Table S1.** Correlations of cell line pairs with respect to their CNV, mutation and pathway activity profiles, as well as their response to all tested drugs in the GDSC.

**Additional file 2: Table S2.** Associations between the expression level of PI3K pathway genes and PI3K inhibitor responses predicted by ML-CDN2. This table lists the PI3K pathway genes which show significant association between their expression levels with the predicted IC50 values of the PI3K pathway inhibitors.

**Additional file 3: Figure S1.** Correlations of cell line pairs, tumor pairs and cell line-tumor pairs. (a) The pairwise Pearson correlation of the 49 BC cell lines from GDSC was calculated based on their gene expression profiles. (b) The pairwise Pearson correlation of the 1100 BC samples from TCGA was calculated based their gene expression profiles. (c) The pairwise Pearson correlation between the 49 BC cell lines and the 1100 BC samples was calculated based their gene expression profiles. (d) The pairwise Pearson correlation of the 49 BC cell lines was calculated based on their pathway activity profiles. (e) The pairwise Pearson correlation of the 1100 BC samples from TCGA was calculated based their pathway activity profiles. (f) The pairwise Pearson correlation between the 49 BC cell lines and the 1100 BC samples was calculated based their pathway activity profiles.

## Data Availability

Gene expression, mutation, and CNV data along with drug response measures for the GDSC dataset are available from the website (ftp://ftp.sanger.ac.uk/pub4/cancerrxgene/releases/release-7.0). Gene expression levels and drug response measures for the CCLE dataset are available from the website (https://portals.broadinstitute.org/ccle).

## Abbreviations

BC: Breast cancer (BC)
CCLE: Cancer Cell Line Encyclopedia
CNV: Copy number variation
CSN: Cell line similarity network
DSN: Drug similarity network
ML-CDN: Multiple-layer cell line-drug response network
GDSC: Genomics of Drug Sensitivity in Cancer
TCGA: The Cancer Genome Atlas
