# Supplementary figures and images for: Predicting breast cancer drug response using a multiple-layer cell line drug response network model
Source: BMC Cancer. 2021 May 31;21:648. doi: 10.1186/s12885-021-08359-6 (PMC8166022; doi:10.1186/s12885-021-08359-6)

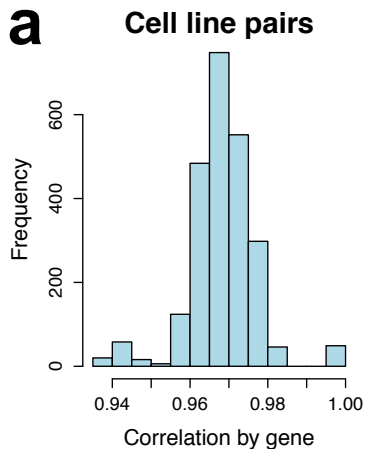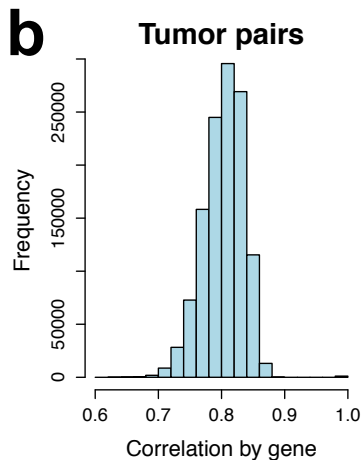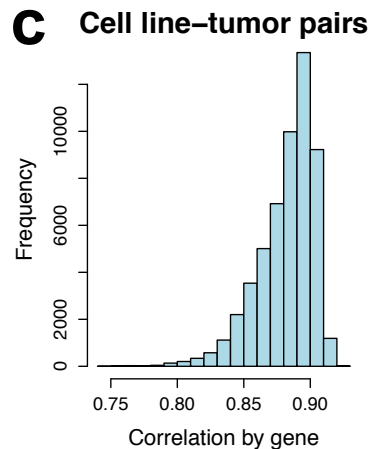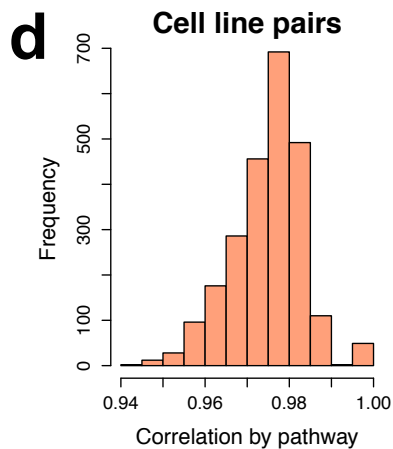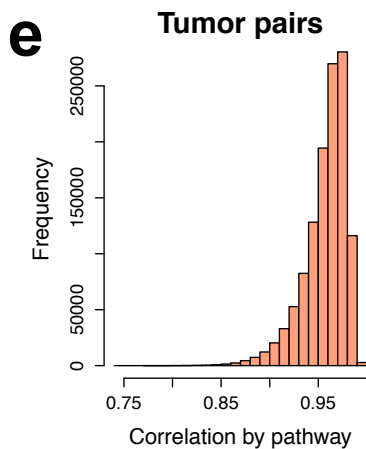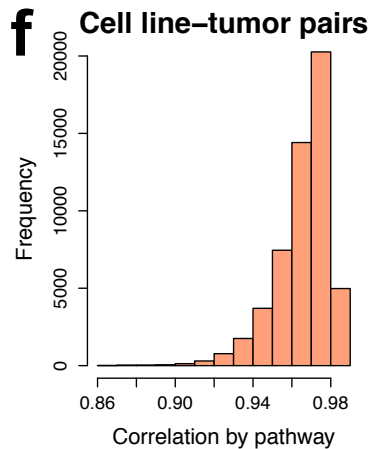

Supplement: Supplementary file 3 — Additional file 3: Figure S1. Correlations of cell line pairs, tumor pairs and cell line-tumor pairs. (a) The pairwise Pearson correlation of the 49 BC cell lines from GDSC was calculated based on their gene expression profiles. (b) The pairwise Pearson correlation of the 1100 BC samples from TCGA was calculated based their gene expression profiles. (c) The pairwise Pearson correlation between the 49 BC cell lines and the 1100 BC samples was calculated based their gene expression profiles. (d) The pairwise Pearson correlation of the 49 BC cell lines was calculated based on their pathway activity profiles. (e) The pairwise Pearson correlation of the 1100 BC samples from TCGA was calculated based their pathway activity profiles. (f) The pairwise Pearson correlation between the 49 BC cell lines and the 1100 BC samples was calculated based their pathway activity profiles. [file 12885_2021_8359_MOESM3_ESM.pdf]
